# Updates in Structural Cardiovascular Interventions: Key Insights from Recent Studies

**DOI:** 10.3390/jcm13206115

**Published:** 2024-10-14

**Authors:** Ana Paula Tagliari, Maurizio Taramasso

**Affiliations:** 1 Instituto de Ciências Básicas da Saúde, Universidade Federal do Rio Grande do Sul, Porto Alegre 90035-003, Brazil; 2 Cardiovascular Surgery Department, Hospital Mãe de Deus, Porto Alegre 90880-0481, Brazil; 3 Clinic of Cardiac Surgery, HerzZentrum Hirslanden Zurich, 8008 Zurich, Switzerland; m.taramasso@gmail.com

The year 2024 brought remarkable advancements and high-quality evidence to the field of cardiovascular interventions. Numerous meta-analyses, randomized clinical trials (RCTs), and cohort studies have provided the cardiovascular community with crucial insights into the safety and efficacy of structural heart valve interventions, such as transcatheter aortic valve intervention (TAVI) and mitral and tricuspid transcatheter edge-to-edge repair (TEER).

In the aortic valve intervention arena, the NOTION 10-year study delivered the longest follow-up of TAVI compared to surgical aortic valve replacement (SAVR), demonstrating not only the durability of transcatheter heart valves (THV) but also its excellent hemodynamic profile, with lower rates of both structural and non-structural valve deterioration compared with SAVR [1]. NOTION 2 was the first RCT to include patients with bicuspid aortic valves. It found that, while TAVI and SAVR showed similar outcomes in patients with tricuspid valves, SAVR remained superior to TAVI regarding mortality, stroke, and rehospitalization in those with bicuspid phenotypes [2]. In turn, NOTION 3 highlighted the advantages of combined percutaneous coronary intervention in patients with symptomatic aortic stenosis and concomitant stable coronary artery lesions (a fractional flow reserve of 0.80 or less or a diameter stenosis of at least 90%) [3]. Further insights into TAVI management were provided by the SMART trial, which evaluated two different THV platforms for approaching small aortic annuli (aortic-valve annulus area of 430 mm^2^ or less) in a predominantly female population. The study demonstrated that the supra-annular self-expanding Evolut platform was clinically and hemodynamically superior to the intra-annular balloon-expandable Sapien one [4]. Lastly, the RHEIA trial, the first RCT exclusively involving women, demonstrated not only the non-inferiority but also the superiority of TAVI over SAVR for a composite endpoint of all-cause mortality, stroke, or rehospitalization for valve- or procedure-related symptoms, or worsening of heart failure at one year [5]. Additionally, the POPular PAUSE study suggested that oral anticoagulation (primarily due to atrial fibrillation) descontinuation before TAVI was better than a continued anticoagulation strategy in terms of a composite outcomes, including cardiovascular death, stroke, myocardial infarction, major vascular complications, or major bleeding, a result mainly driven by a lower bleeding rate in the interruption group [6].

The mitral valve field also presented significant progress, with two major RCTs addressing secondary mitral regurgitation (SMR). The RESHAPE-HF2 trial aimed to address the gap left by the previous COAPT and MITRA-FR trials by evaluating patients with SMR and moderate to severe MR. The intervention group experienced significantly fewer first or recurrent heart failure hospitalizations compared to those receiving medical therapy alone, although with no benefit in terms of mortality rates at 24 months [7]. The MATTERHORN trial innovatively demonstrated that in patients with severe SMR and with no surgical contraindication, mitral transcatheter edge-to-edge repair (TEER) was non-inferior to mitral valve surgery (repair or replacement) in terms of death, hospitalization for heart failure, mitral-valve reintervention, implantation of an assist device, or stroke within 1 year after the procedure [8].

Last but not least, the tricuspid valve was explored in the TRIGISTRY and TRI-FR studies. The former showed a notable long-term follow-up (up to 10 years) survival benefit in lower-risk patients (as assessed by the TRI-SCORE) with severe isolated tricuspid regurgitation (TR) who underwent surgical intervention (tricuspid valve repair or replacement) compared to conservative management. Intermediate-risk patients also presented significant survival benefit when submitted to a tricuspid valve repair, a result that was not true with valve replacement, which was even inferior when compared to medical management [9]. The TRI-FR study supported the previous TRILUMINATE trial results [10], confirming the benefits of the TRICLIP therapy in terms of symptom relief, quality of life improvement, and reduction in TR degree, although with no impact on hard outcomes such as heart failure hospitalization rates or mortality [11].

Even though the RCTs mentioned above have garnered significant attention from the cardiovascular community by addressing critical and long-awaited questions, the importance of original cohort studies and real-world data should not be overlooked. With this in mind, the “Transcatheter Structural Heart Disease Interventions: Clinical Update—The Second Edition”, published by the Journal of Clinical Medicine (JCM), gathering four original articles and one review, offers valuable evidence and expert perspectives for readers interested in structural heart disease interventions (Table 1).

To open this special edition, the guest editors discuss, in a how-to article, their current approach to performing TAVI procedures, emphasizing a minimally invasive approach and providing tips and tricks to minimize invasiveness while maintaining TAVI safety and effectiveness [12].

Four additional original studies explored various aspects of structural heart disease interventions. In the first, an Italian research group evaluated the closure of patent foramen ovale (PFO) using the Gore Septal Occluder device, demonstrating how different PFO anatomies affect device performance and procedural effectiveness. In the second, a systematic review and meta-analysis, Balakrishna et al. investigated the incidence of infective endocarditis (IE) following transcatheter pulmonary valve implantation with either the Medtronic Melody or Edwards SAPIEN THV. The analysis revealed a pooled IE incidence of 2.1% (95% CI: 0.9% to 5.13%) for Sapien valves compared to 8.5% (95% CI: 4.8% to 15.2%) for Melody valves, indicating a 79.6% lower risk of IE with the SAPIEN valve. In the third, Fernandez-Peregrina et al. investigated the prognostic value of left ventricle global longitudinal strain (LVGLS) in patients with reduced left ventricle function (LVEF ≤ 40%) submitted to mitral TEER. Their retrospective analysis identified LVGLS as an independent predictor of cardiovascular mortality, particularly in those with a more depressed LVEF (<30%) (HR: 3.3; 95% CI: 1.1–10, *p* = 0.023). Finally, patients from the France-TAVI Registry were evaluated regarding the effects of paravalvular leak (PVL) and patient–prosthesis mismatch (PPM) on TAVI outcomes. Among 47,494 patients, PPM was observed in 29.0% and PVL ≥ 2 in 19.4% of the cohort. Data showed that, whereas PPM was not associated with an increased risk of mortality, PVL ≥ 2 was an independent predictor of mortality at 6.5 years (Table 1).

In summary, the articles presented in this Special Issue complement the pool of strong and high-level evidence generated over this year. They provide a comprehensive overview of transcatheter heart interventions, offering valuable insights and enhancing our understanding of the rapidly evolving field of cardiovascular interventions.

## Figures and Tables

**Table 1:** Summary of the papers published in the Special Issue “Transcatheter Structural Heart Disease Interventions: Clinical Update—The Second Edition”.

Contribution Number	Reference	Type of Publication	Number of Authors/ Affiliations	Locations of Authors’ Affiliations
1	Tagliari, A.P.; Taramasso, M. New Practices in Transcatheter Aortic Valve Implantation: How I Do It in 2023. J. Clin. Med. 2023, 12, 1342.	How I Do It	2/3	Brazil, Switzerland
2	Verolino, G.; Calderone, D.; Gavazzoni, M.; Sala, D.; Sganzerla, P. Clinical Performance of the Gore Septal Occluder in Patent Foramen Ovale Closure in Different Septal Anatomies: 1-Year Results from a Single-Center Experience. J. Clin. Med. 2023, 12, 5936.	Original Article	5/2	Italy
3	Machanahalli Balakrishna, A.; Dilsaver, D.B.; Aboeata, A.; Gowda, R.M.; Goldsweig, A.M.; Vallabhajosyula, S.; Anderson, J.H.; Simard, T.; Jhand, A. Infective Endocarditis Risk with Melody versus Sapien Valves Following Transcatheter Pulmonary Valve Implantation: A Systematic Review and Meta-Analysis of Prospective Cohort Studies. J. Clin. Med. 2023, 12, 4886.	Systematic Review and Meta-Analysis	9/8	United States of America
4	Fernandez-Peregrina, E.; Asmarats, L.; Estevez-Loureiro, R.; Pascual, I.; Bastidas, D.; Benito-González, T.; Caneiro-Queija, B.; Avanzas, P.; De Agustin, J.A.; Fernández-Vazquez, F.; et al. Global Longitudinal Strain Predicts Outcomes in Patients with Reduced Left Ventricular Function Undergoing Transcatheter Edge-to-Edge Mitral Repair. J. Clin. Med. 2023, 12, 4116.	Original Article	20/7	Spain
5	Deharo, P.; Leroux, L.; Theron, A.; Ferrara, J.; Vaillier, A.; Jaussaud, N.; Porto, A.; Morera, P.; Gariboldi, V.; Iung, B.; et al. Long-Term Prognosis Value of Paravalvular Leak and Patient–Prosthesis Mismatch following Transcatheter Aortic Valve Implantation: Insight from the France-TAVI Registry. J. Clin. Med. 2022, 11, 6117.	Original Article	27/17	France
